# Identifying the Factors That Affect Depressive Symptoms in Middle-Aged Menopausal Women: A Nationwide Study in Korea

**DOI:** 10.3390/ijerph17228505

**Published:** 2020-11-17

**Authors:** Kisook Kim

**Affiliations:** Department of Nursing, Chung-Ang University, Seoul 06974, Korea; kiskim@cau.ac.kr

**Keywords:** middle-aged, postmenopausal, depressive symptom, women

## Abstract

Depressive symptoms do not persistently negatively affect functioning throughout the life span, but they may affect quality of life (QOL), especially in middle-aged women. Therefore, this study aimed to investigate the factors associated with depressive symptoms in premenopausal and postmenopausal women. This descriptive study was a secondary analysis of the data from the seventh Korea National Health and Nutrition Examination Survey that was conducted in 2016. Of the 8150 participants, the analysis included 644 premenopausal women and 459 postmenopausal women aged 40–60 years. The factors associated with depressive symptoms in the participants were examined using multivariate regression analysis. It was found that the depressive symptoms of postmenopausal middle-aged women were greater than those of premenopausal women. While a high perceived stress status and high pain-related discomfort were factors that influenced depressive symptoms among the premenopausal group, a high level of discomfort due to anxiety/depression, a poor perceived health status, shortened weekend sleep time, and smoking were associated with depressive symptoms in the postmenopausal group. Thus, it is necessary to develop an intervention to decrease depressive symptoms in postmenopausal women that considers their QOL, which is associated with depression, their subjective health status, sleep time, and smoking cessation.

## 1. Introduction

Depression affects not only an individual’s mental health but also his/her physical health and it has a negative effect on health-related quality of life (QOL) [1,2]. Depression is common and it can have a significant impact on individuals, families, the workplace, and the healthcare system [3]. Depressive symptoms are part of depressive disorders, but they may not persistently negatively affect functioning throughout the life span. However, QOL may be affected by the presence of depressive symptoms even in the absence of a depressive disorder [3,4]. Several studies have found that depressive symptoms, which can be measured using various depression scales, are positively associated with menopause transition and the postmenopausal period independent of the risk factors for depressive disorder [5,6,7,8].

Middle-aged women’s lives are characterized by physiological and psychological changes that can affect various dimensions of life [9]. Menopause is a normal degenerative transition that is associated with aging and the loss of reproductive function, and menopausal women experience not only biological changes but also social and cultural changes [10]. These changes during the transition make menopausal women more vulnerable to physical and mental health problems [11,12]. Physical function is lowered as chronic diseases progress along with the presence of hormonal imbalances, decreased adaptive capacity, and physical aging [9]. Their psychological health status is also worse than in the premenopausal period, and postmenopausal women feel that their lives are meaningless [13]. Furthermore, changes in female hormones often lead to mental problems, such as somatization symptoms and depression, in postmenopausal women, which in turn reduces QOL [14,15,16].

Those who enter the menopause transition early are twice as likely to develop severe depressive symptoms [17]. In addition, one study has reported that 24% of middle-aged women have depressive symptoms that correspond to major depression [18], and another has found that 22–29% of naturally menopausal women report psychologic distress during the perimenopausal period [19].

Furthermore, symptoms of anxiety and depression are common in midlife and they may coincide with menopausal symptoms [8]. Menopause is also associated with depressive symptoms that can significantly affect a woman’s QOL [20]. In addition, assuming that the average life expectancy of women in developed countries is 84.3 years and that the average age at menopause is 50 years, the postmenopausal period accounts for almost half or a third of women’s lives [21].

Hormone replacement therapy is considered the best option for achieving the therapeutic relief of various menopausal symptoms, but there are various restrictions, such as administration conditions, and there are many concerns about side effects [22]. Thus, studies of the nutraceutical approach are being conducted to examine whether nutraceuticals reduce menopausal complaints, and non-hormonal treatments, such as pollen extracts and soy isoflavones, can be effectively used in menopausal women who have hot flashes, sleep disturbances, and menopausal symptoms [23,24].

Various studies have also been conducted to identify the factors that affect depressive symptoms in postmenopausal women [6,8,20,25]. However, these previous studies have focused on longitudinal changes in depressive symptoms, the management of depressive symptoms, hormones, and the factors that affect depressive symptoms in older menopausal women. No studies have compared the factors that influence depressive symptoms between postmenopausal women and premenopausal women. In addition, the Patient Health Questionnaire-9 (PHQ-9) was first used in the sixth Korean national survey in 2014, and it was employed in the seventh survey (2016–2018) to screen for depression and to determine the depressive symptom statistics of the Korean population [26].

Therefore, it is necessary to investigate the various factors that are related to depressive symptoms that result from the menopause among middle-aged women, by using a large sample and a representative population. In this study, we investigated the factors that are particularly associated with depressive symptoms in postmenopausal women by comparing them between premenopausal and postmenopausal women.

## 2. Materials and Methods

### 2.1. Study Design

This study was a descriptive cross-sectional study. It aimed to investigate the factors that affect depressive symptoms among middle-aged women based on the menopause occurrence. This study was conducted according to the Strengthening the Reporting of Observational Studies in Epidemiology statement’s reporting guidelines [27].

### 2.2. Study Population and Data Source

This study used data from the seventh Korea National Health and Nutrition Examination Survey (KNHANES VII-1) that was conducted in 2016, which is a nationally representative survey that is undertaken by the Korea Centers for Disease Control and Prevention (KCDC). The KNHANES VII-1 involved a stratified sample of 8150 participants that was based on various inherent strata (e.g., sex, age, and residence size) and depended on the residential area, and it comprised composite samples of 4416 households in 192 survey groups nationwide. Twenty-three final survey households per enumeration district were extracted from the sample enumeration districts using a systematic sampling method. The participants were people aged ≥1 year who lived in the extracted households. The participation rate was 75.4%. Each participant provided written informed consent before taking part.

Our analysis included 644 premenopausal women and 459 postmenopausal women from the survey who were aged 40–60 years. The postmenopausal women were naturally menopausal. We excluded women who had an artificial menopause (e.g., secondary to hormone therapy, chemotherapy, or surgery).

### 2.3. Measures

#### 2.3.1. Depressive Symptoms

The depressive symptoms of the middle-aged women were measured using the PHQ-9. The PHQ-9 is a nine-item self-reported questionnaire that was developed to diagnose mental illnesses, and it is easy to use in everyday situations. The participants are asked to choose between 0 and 3 points for each item according to the severity of their symptoms, and the total possible score ranges from 0 to 27. The higher the score, the more severe the depressive symptoms. The cutoff point for depression is 10 out of 27. This questionnaire, which has fewer questions and takes less time to complete than the existing depression screening scales, has been used as the primary testing tool for depression screening in a number of countries [28]. The reliability and validity of the Korean version have been previously verified [29].

#### 2.3.2. Demographic Characteristics and the Women’s Health

The demographic characteristics of the participants that were analyzed included age, education level, number of family members, occupation status, average working hours (per week), shift duty, and monthly household income. In addition, breastfeeding experience, total breastfeeding duration, birth experience, number of pregnancies, age at first and last births, and age at menopause (in the case of postmenopausal women) were included for the women’s health factors.

#### 2.3.3. Current Health Status and Health Behaviors

With regard to the participants’ current health-related factors, health-related QOL (HRQOL; which was assessed using the EuroQol 5-Dimension (EQ-5D) questionnaire), perceived health status, perceived stress status, body mass index (BMI), change in body weight (more than a 3 kg change over the past year), number of days of illness (per year), and number of hospital admissions (per year) were included. The EQ-5D is an effective international comparison tool for simple HRQOL assessment [30]. It measures participants’ HRQOL with regard to mobility, self-care, usual activities, pain/disability, and anxiety/depression. Each question has three possible responses that express the current state (1 = no problems, 2 = some problems, and 3 = extreme problems), and the total score (the EQ-5D index) is calculated using a quality weight model that was developed to present the weight of the Korean QOL [30,31]. In general, each dimension of the EQ-5D is considered to show higher discomfort as the score increases, and the higher the score of the EQ-5D index, the higher the overall HRQOL.

The participants’ perceived health status was determined using a Likert-type scale response to the question “What do you think about your usual health?” and it was rated from 1 (very good) to 5 (very bad). The perceived stress status was evaluated using the question “How often do you feel stress in your daily life?” and it was rated from 1 (very often) to 4 (almost never). Regarding health behaviors, the number of days of walking (at least 10 min in one week), number of days of performing strength exercises (in one week), sleep time (minutes per day), smoking status, and alcohol consumption status were included.

#### 2.3.4. Ethical Considerations

The use of the original data from the KNHANES in this study adheres to the personal information protection law and statistical law, and it provides only data that cannot be estimated from the survey data. The researcher applied for the required information on the KCDC website before starting the study. In addition, the researcher downloaded the raw data after receiving approval to use the material (https://knhanes.cdc.go.kr/).

#### 2.3.5. Statistical Analysis

The data were analyzed using SPSS Statistics for Windows version 23.0 (IBM Corp., Armonk, NY, USA). The general characteristics and main variables of the participants were analyzed using descriptive statistics. Depressive-symptom-related variables and the general characteristics of the participants were examined using *t*-tests and χ^2^ tests. The relationship between the depressive symptoms and each variable was determined by conducting a correlation analysis. Multivariate linear regression was performed to investigate the factors that affect depressive symptoms among middle-aged women according to their menopause status. The variance inflation factor (VIF) and Durbin–Watson value were calculated to confirm the multicollinearity between the independent variables, and the independent predictive variables for depressive symptoms that result from menopause were determined. Following the correlation analysis, the variables that were found to be significantly associated with depressive symptoms (*p* < 0.01) in each group were included in the regression analysis. The fit of the multivariate linear regression modeling was confirmed by calculating the adjusted *R*^2^.

## 3. Results

### 3.1. Depressive Symptoms and Demographic Characteristics

The PHQ-9 scores for the depressive symptoms were 2.33 in the premenopausal group and 2.86 in the postmenopausal group, which indicated that the postmenopausal group had higher scores than the premenopausal group (*t* = 2.483, *p* = 0.013).

With regard to the demographic characteristics, the mean age of the premenopausal group was 45.52 years, and it was 54.85 years in the postmenopausal group (*t* = 43.820, *p* < 0.001). The age of the women in the premenopausal group ranged between 40 and 59 years, and the range in the postmenopausal group was 45 to 60 years. Regarding the premenopausal and postmenopausal groups, the proportion of women with a college level education was 45.3% and 21.1% (χ^2^ = 149.04, *p* < 0.001), the monthly household income was 5145.1 and 4642.2 USD (*t* = 2.661, *p* = 0.008), the proportion of women living with their spouses was 91% and 83.6% (χ^2^ = 13.390, *p* < 0.001), and the mean number of women living with family members was 3.38 and 2.84 (*t* = 8.167, *p* < 0.001), respectively. With regard to occupation, 62.3% and 60.8% of women in the premenopausal and postmenopausal groups were employed, their average working hours per week were 37.59 and 41.79, and 15.9% and 20.4% worked in shifts, respectively.

In terms of their health condition, breastfeeding experience was reported by 77% and 81.5% of women in the premenopausal and postmenopausal groups, with a total breastfeeding duration of 3.78 and 3.01 months (*t* = 3.300, *p* = 0.001), respectively. Furthermore, 98.5% and 98.7% of participants had experience of giving birth in the premenopausal and postmenopausal groups, respectively. In addition, the mean age when having the first baby in each group was 26.97 and 24.74 years (*t* = 8.900, *p* < 0.001), the mean age at the last birth was 30.31 and 28.52 years, and the number of pregnancies was 3.21 and 3.92 (*t* = 7.335, *p* < 0.001) in the premenopausal and postmenopausal groups, respectively (Table 1).

### 3.2. Current Health Status and Health Behaviors

For the HRQOL items, the scores for activity, self-control, usual care, pain/discomfort, and anxiety/depression were higher in the postmenopausal group, and the index was higher in the premenopausal group. Among the EQ-5D items, activity (*t* = 4.587, *p* < 0.001), pain/discomfort (*t* = 3.779, *p* < 0.001), and the index (*t* = 4.260, *p* < 0.001) were statistically significant. In other words, the postmenopausal group showed worse activity, pain/discomfort, and overall QOL scores than the premenopausal group. In addition, the perceived health status was better in the premenopausal group (*t* = 2.721, *p* = 0.007), and the BMI was higher in the postmenopausal group (*t* = 2.825, *p* = 0.005). Regarding the change in weight, 32.5% and 25.5% of the premenopausal and postmenopausal women, respectively, answered that they had experienced a weight gain of more than 3 kg in the past year (*t* = 8.210, *p* = 0.016). Weekend sleep times in the premenopausal and postmenopausal groups were 468.48 and 437.53 min (*t* = 6.229, *p* < 0.001), respectively. With regard to the health behaviors, the results of the smoking, drinking, days of walking, and days of strength exercises are shown in Table 2.

### 3.3. Relationship between Depressive Symptoms and the Study Variables

There were statistically significant correlations between the depressive symptoms and the education level (*r* = −0.197, *p* < 0.001), income (*r* = −0.211, *p* < 0.001), number of family members living together (*r* = −0.122, *p* = 0.002), and shift duty (*r* = −0.126, *p* = 0.008) in the premenopausal group. In other words, in terms of the demographic characteristics, the depressive symptom scores were higher in the women who had a low level of education, low income, and fewer family members, and who worked night shifts. In addition, regarding the current health status, the EQ-5D index (*r* = −0.506, *p* = 0.001), perceived health status (*r* = 0.388, *p* < 0.001), perceived stress status (*r* = −0.437, *p* < 0.001), change in body weight (*r* = 0.089, *p* = 0.024), number of days of illness (*r* = 0.262, *p* = 0.003), and number of hospital admissions (r = −0.106, *p* = 0.007) showed a statistically significant correlation with the depressive symptom scores. Thus, a high score for the depressive symptoms was related to low HRQOL, poor perceived health status, high perceived stress status, weight gain, a large increase in the number of days of illness, and fewer hospital admissions.

In the postmenopausal group, there were statistically significant correlations between the depressive symptoms and the education level (*r* = −0.135, *p* = 0.004) and income (*r* = −0.205, *p* < 0.001) in terms of the demographic characteristics, and the total breastfeeding duration (*r* = −0.123, *p* = 0.020) and number of pregnancies (*r* = 0.135, *p* = 0.004) in terms of the women’s health. This meant that the depressive symptoms were also positively correlated with a low level of education, low income, short breastfeeding duration, and high pregnancy count. In addition, in terms of the current health status, the EQ-5D index (*r* = −0.509, *p* < 0.001), perceived health status (*r* = 0.429, *p* < 0.001), perceived stress status (*r* = −0.430, *p* < 0.001), and number of days of illness (*r* = 0.367, *p* < 0.001) showed statistically significant correlations with the depressive symptom scores. Unlike the premenopausal group’s results, the postmenopausal group’s results showed that there were significant correlations between the depressive symptoms and days of walking (*r* = −0.129, *p* = 0.019), weekday sleep time (*r* = −0.099, *p* = 0.034), weekend sleep time (*r* = −0.123, *p* = 0.008), and smoking status (*r* = 0.329, *p* < 0.001). Thus, depressive symptoms were positively correlated with fewer days of walking, lower sleep time, and smoking (Table 3).

### 3.4. Factors Associated with the Depressive Symptoms According to Menopause

To investigate the factors that affect the depressive symptoms of middle-aged women according to their menopause status, multivariate regression analysis was performed, and the VIF ranges were found to be 1.025 and 1.162, and the Durbin–Watson values were 1.889 and 1.853 in the premenopausal and postmenopausal groups, respectively. Thus, the possibility of multicollinearity could be ruled out. Following the correlation analysis, the variables that were significantly associated with the depressive symptoms (*p* < 0.01) in each group were included in the regression analysis. The adjusted *R*^2^ values were used for the fit of the regression model. Education, income, living with family members, shift duty (premenopausal group), age at last childbirth (premenopausal group), breastfeeding duration (postmenopausal group), number of pregnancies (postmenopausal group), days of walking (postmenopausal group), days of strength exercises (postmenopausal group), smoking, sleep duration (postmenopausal group), drinking (premenopausal group), the EQ-5D, perceived health status, perceived stress status, BMI (premenopausal group), change in body weight (premenopausal group), days of illness, and number of hospital admissions were included in the analysis.

The multiple regression analysis revealed that perceived stress status (*t* = −3.652, *p* = 0.001) and the EQ-5D’s pain/discomfort (*t* = 3.109, *p* = 0.003) accounted for 30.7% of the variance in the depressive symptoms of the premenopausal women (adjusted *R*^2^ = 0.307, *F* = 13.620, *p* < 0.001). In addition, the EQ-5D’s anxiety/depression (*t* = 3.891, *p* < 0.001), perceived health status (*t* = 2.880, *p* = 0.005), sleep time (especially at the weekend; *t* = −2.646, *p* = 0.010), and smoking (*t* = 2.398, *p* = 0.020) were factors that affected depressive symptoms among the postmenopausal women, and they accounted for 46.8% of the variance in the depressive symptoms (adjusted *R*^2^ = 0.468, *F* = 15.310, *p* < 0.001) (Table 4).

To summarize, depressive symptoms were associated with factors such as a higher level of stress and pain/discomfort among the group of premenopausal women. In addition, a high level of discomfort related to anxiety/depression, poor perceived health status, low sleep time at the weekend, and smoking were associated with depressive symptoms in the postmenopausal women.

## 4. Discussion

This study aimed to identify the factors that are associated with depressive symptoms before and after menopause in middle-aged women. According to the study, the depressive symptoms of postmenopausal middle-aged women were greater than those of premenopausal women. While a high level of perceived stress and high level of pain-related discomfort were factors that influenced depressive symptoms among the premenopausal women, discomfort due to a high level of anxiety/depression, poor perceived health status, shortened weekend sleep time, and smoking were associated with depressive symptoms in the postmenopausal women.

The risk of depressive symptoms is almost 2–3 times higher in women who are in the menopause transition than in premenopausal women [8,32]. Postmenopausal women undergo considerable biological and psychological changes, including a decrease in estrogen levels, which may be associated with depressive symptoms [32,33,34]. These depressive symptoms, rather than other menopausal symptoms, have an important impact on postmenopausal women’s QOL [35,36]. In addition, changes in female hormones often cause mental problems, such as somatization symptoms and depressive symptoms in postmenopausal women, which also reduce QOL [14,15,16]. Furthermore, the significant predictors of QOL for perimenopausal women are depressive symptoms, self-reported health status, menopausal symptoms, education level, and marital status [37]. Therefore, various interventions should be considered to improve the QOL of postmenopausal women, including participation in physical exercise, appropriate hormone therapy, and education and counseling programs [38].

One of the prognostic indicators of depressive symptoms is self-rated health. People who report poor health are more likely to have depression than those who report good health [39]. In addition, perceived health status has a synergetic effect on depressive symptoms among middle-aged and elderly individuals through its interaction with social activity [40]. However, postmenopausal women have a poorer perceived health status than premenopausal women; therefore, it is necessary to consider providing interventions to improve the depressive symptoms that take these related factors into account.

Light and interrupted sleep is a common symptom in postmenopausal women [41], and perimenopausal women also have relatively poor sleep quality [42]. In particular, postmenopausal women tend to have a higher BMI and central adiposity [43], and these factors are strongly associated with breathing-related sleep disturbances [44]. Furthermore, sleep quality predicts the depressive symptoms of menopausal women [45], and various menopausal symptoms can potentially influence a depressed mood through sleep disturbance [46]. Because 60.8% of postmenopausal women in this study had an occupation, it is necessary to identify an intervention that can control depressive symptoms via adequate weekend rest and the improvement of sleep quality. Cognitive behavioral therapy for insomnia and sleep restriction therapy could be efficacious options for postmenopausal women who have chronic sleep disturbance [47].

In particular, smoking is a risk factor for vasomotor and depressive symptoms during the menopause transition [48] as well as other demographic and therapeutic factors. That the cessation of smoking is a change in lifestyle that can prevent postmenopausal disease is an important finding [49]. Therefore, based on the findings of this study, it is necessary to develop an intervention to reduce depressive symptoms in postmenopausal women that considers the QOL associated with depression, subjective health status, sleeping time, and smoking cessation.

### Strengths and Limitations

This study has the following strengths. Above all, it is the first large-scale national study in Korea to investigate the factors that affect depressive symptoms among postmenopausal women that uses the PHQ-9, which is a specific depressive symptom measurement tool with established validity and reliability. Furthermore, the present study provides the basis for the development of specific interventions by presenting the factors that influence depressive symptoms pre- and post-menopause. However, there are also several limitations to this study. The participants who were in menopause transition were not distinguished clearly due to the limits of the large-scale survey. Vasomotor symptoms, such as hot flashes, which can affect the depressive symptoms of postmenopausal women, were also not considered in this study due to the limitation of it involving secondary data analysis. In addition, there was no consideration of hormone therapy, which is known to be effective for various menopausal symptoms, including depressive symptoms. Furthermore, participants with a diagnosis of depression may have been included, and they could not be excluded and analyzed. Therefore, there is a limit to the generalizability of this study’s results.

## Figures and Tables

**Table 1:** Depressive symptoms, demographic characteristics, and women’s health factors according to the menopause.

		Premenopausal Group (*n* = 644)	Postmenopausal Group (*n* = 459)	*t* or χ^2^	*p*
		Mean (*SD*)/*N* (%)			
Depressive symptoms					
PHQ-9		2.33 (3.14)	2.86 (3.99)	2.483	0.013
Demographic characteristics					
Age		45.52 (3.73)	54.85 (3.11)	43.820	<0.001
Education	Elementary	23 (3.6)	81 (17.6)	149.04	<0.001
	Middle	36 (5.6)	91 (19.8)		
	High	293 (45.5)	190 (41.4)		
	≥College	292 (45.3)	97 (21.1)		
Monthly household income (USD)		5145.1 (2999.02)	4642.2 (3217.59)	2.661	0.008
Living with spouse	Yes	565 (91.0)	377 (83.6)	13.390	<0.001
	No	56 (9.0)	74 (16.4)		
Living with family members		3.38 (1.09)	2.84 (1.05)	8.167	<0.0001
Occupation	Have	401 (62.3)	279 (60.8)	0.249	0.660
	None	243 (37.3)	180 (39.2)		
Working hours (average/week)		37.59 (16.06)	41.79 (56.84)	1.474	0.141
Shift duty	Day duty	371 (84.1)	250 (79.6)		
	Shift duty	70 (15.9)	64 (20.4)		
Women’s health factors					
Breastfeeding experience (more than 1 month)	Yes	469 (77.0)	361 (81.5)	3.089	0.092
	No	140 (23.0)	82 (18.5)		
Total breastfeeding duration (months)		3.78 (3.27)	3.01 (3.41)	3.300	0.001
Birth experience	Yes	609 (98.5)	443 (98.7)	0.27	1.00
	No	9 (1.5)	6 (1.3)		
Age at first birth (years)		26.97 (4.24)	24.74 (3.67)	8.900	<0.001
Age at last birth (years)		30.31 (4.51)	28.52 (4.29)	1.796	0.073
Age at menopause (years)		-	50.01 (3.34)	-	
Number of pregnancies		3.21 (1.44)	3.92 (1.69)	7.335	<0.001

PHQ-9, Patient Health Questionnaire-9.

**Table 2:** Current health status and health behaviors according to the menopause.

		Premenopausal Group (*n* = 644)	Postmenopausal Group (*n* = 459)	*t* or χ^2^	*p*
		Mean (*SD*)/*N* (%)			
Current health status					
EQ-5D	Activity	1.05 (0.22)	1.12 (0.33)	4.587	<0.001
	Self-control	1.01 (0.11)	1.02 (0.14)	1.365	0.172
	Usual care	1.03 (0.19)	1.05 (0.21)	1.773	0.760
	Pain/discomfort	1.17 (0.39)	1.27 (0.49)	3.779	<0.001
	Anxiety/depression	1.08 (0.28)	1.13 (0.35)	2.529	0.12
	EQ-5D index	0.97 (0.07)	0.95 (0.09)	4.260	<0.001
Perceived health status		2.82 (0.77)	2.95 (0.85)	2.721	0.007
Perceived stress status		2.83 (0.68)	2.83 (0.71)	0.177	0.860
BMI		23.39 (3.60)	24.00 (3.46)	2.825	0.005
Change in body weight	No change	389 (60.8)	296 (64.6)	8.210	0.016
	Loss	43 (6.7)	45 (9.8)		
	Gain	208 (32.5)	117 (25.5)		
Days of illness (days/year)		10.38 (17.59)	19.01 (50.19)	1.836	0.68
Hospital admissions (count/year)		0.12 (0.42)	0.18 (0.49)	2.142	0.32
Health behaviors					
Days of walking (per week)		3.96 (4.75)	3.67 (1.98)	1.051	0.293
Days of strength exercises (per week)		1.53 (1.26)	1.59 (0.38)	0.722	0.470
Sleep time (min)	Weekend	468.47 (81.99)	437.53 (80.32)	6.229	<0.001
	Weekday	413.94 (76.40)	407.09 (72.47)	1.498	0.134
Smoking	No	584 (91.1)	417 (91.0)	0.001	0.973
	Yes	57 (8.9)	41 (9.0)		
Drinking	No	232 (47.9)	160 (54.6)	3.252	0.071
	Yes	252 (52.1)	133 (45.4)		

EQ-5D, EuroQol 5-Dimension questionnaire; BMI, body mass index.

**Table 3:** Correlations between the depressive symptoms and study variables.

		Premenopausal Group	Postmenopausal Group		Premenopausal Group	Postmenopausal Group
		*r* (*p*)			*r* (*p*)	
Demographic characteristics				Current health status		
Age		0.004 (0.927)	0.011 (0.820)	EQ-5D activity	0.276 (<0.001)	0.404 (<0.001)
Education		−0.197 (<0.001)	−0.135 (0.004)	Self-control	0.244 (<.0001)	0.131 (<0.001)
Monthly household income		−0.211 (<0.001)	−0.205 (<0.001)	Usual care	0.344 (<0.001)	0.248 (<0.001)
Living with family members		−0.122 (0.002)	−0.083 (0.075)	Pain/discomfort	0.357 (<0.001)	0.378 (<0.001)
Working hours		0.070 (0.140)	−0.001 (0.992)	Anxiety/depression	0.470 (<0.001)	0.473 (<0.001)
Shift duty		−0.126 (0.008)	−0.006 (0.920)	Index	−0.506 (0.001)	−0.509 (<0.001)
Women’s health factors				Perceived health status	0.388 (<0.001)	0.429 (<0.001)
Breastfeeding duration		0.043 (0.355)	−0.123 (0.020)	Perceived stress status	−0.437 (<0.001)	−0.430 (<0.001)
Age at first birth		−0.058 (0.153)	−0.028 (0.552)	BMI	0.072 (0.070)	−0.033 (0.482)
Age at last birth		−0.076 (0.061)	−0.045 (0.344)	Change in body weight	0.089 (0.024)	0.059 (0.211)
Number of pregnancies		0.032 (0.423)	0.135 (0.004)	Days of illness	0.262 (0.003)	0.367 (<0.001)
Age at menopause		-	−0.043 (0.362)	Hospital admissions	−0.106 (0.007)	−0.081 (0.084)
Menopause period		-	0.046 (0.321)			
Health behaviors						
Days of walking		−0.073 (0.110)	−0.129 (0.019)	Sleep time (weekday)	−0.037 (0.349)	−0.099 (0.034)
Days of strength exercises		−0.010 (0.800)	−0.092 (0.050)	(weekend)	−0.049 (0.213)	−0.123 (0.008)
Smoking		0.071 (0.072)	0.329 (<0.001)	Drinking	0.080 (0.080)	0.086 (0.143)

EQ-5D, EuroQol 5-Dimension questionnaire; BMI, body mass index.

**Table 4:** Factors associated with depressive symptoms according to menopause.

Categories	B	Beta	*t*	*p*	*F*	*p*	*R* ^2^	Adjusted *R*^2^
Premenopausal women								
(Constant)	6.454		2.783	0.007	13.620	<0.001	0.331	0.307
Perceived stress status	−2.483	−0.408	−3.652	0.001				
EQ-5D: pain/discomfort	2.737	0.347	3.109	0.003				
Postmenopausal women								
(Constant)	−0.703		−0.212	0.833	15.310	<0.001	0.501	0.468
EQ-5D: anxiety/depression	3.886	0.377	3.891	<0.001				
Perceived health status	1.750	0.281	2.880	0.005				
Sleep time (weekend)	−0.013	−0.250	−2.646	0.010				
Smoking	3.530	0.230	2.398	0.020				

EQ-5D, EuroQol 5-Dimension questionnaire.
